# uHAF: a unified hierarchical annotation framework for cell type standardization and harmonization

**DOI:** 10.1093/bioinformatics/btaf149

**Published:** 2025-04-02

**Authors:** Haiyang Bian, Yinxin Chen, Lei Wei, Xuegong Zhang

**Affiliations:** MOE Key Laboratory of Bioinformatics and Bioinformatics Division of BNRIST, Department of Automation, Tsinghua University, Beijing 100084, China; MOE Key Laboratory of Bioinformatics and Bioinformatics Division of BNRIST, Department of Automation, Tsinghua University, Beijing 100084, China; MOE Key Laboratory of Bioinformatics and Bioinformatics Division of BNRIST, Department of Automation, Tsinghua University, Beijing 100084, China; MOE Key Laboratory of Bioinformatics and Bioinformatics Division of BNRIST, Department of Automation, Tsinghua University, Beijing 100084, China; Center for Synthetic and Systems Biology, School of Life Sciences and School of Medicine, Tsinghua University, Beijing 100084, China

## Abstract

**Summary:**

In single-cell transcriptomics, inconsistent cell type annotations due to varied naming conventions and hierarchical granularity impede data integration, machine learning applications, and meaningful evaluations. To address this challenge, we developed the unified Hierarchical Annotation Framework (uHAF), which includes organ-specific hierarchical cell type trees (uHAF-T) and a mapping tool (uHAF-Agent) based on large language models. uHAF-T provides standardized hierarchical references for 38 organs, allowing for consistent label unification and analysis at different levels of granularity. uHAF-Agent leverages GPT-4 to accurately map diverse and informal cell type labels onto uHAF-T nodes, streamlining the harmonization process. By simplifying label unification, uHAF enhances data integration, supports machine learning applications, and enables biologically meaningful evaluations of annotation methods. Our framework serves as an essential resource for standardizing cell type annotations and fostering collaborative refinement in the single-cell research community.

**Availability and implementation:**

uHAF is publicly available at: https://uhaf.unifiedcellatlas.org and https://github.com/SuperBianC/uhaf.

## 1 Introduction

Over the past decade, the rapidly advancing field of single-cell transcriptomics has generated an enormous and ever-increasing volume of datasets. In single-cell biology, cell type labels are the most crucial attributes of cells, assigned by various institutions and researchers involved in data analysis. However, due to the multitude of data sources, the same cell type is often annotated with different names or at varying levels of hierarchical granularity by different researchers. For example, one researcher might abbreviate “cardiomyocyte” as “CM,” while another might use the full term “cardiomyocyte” or refer to it as “heart muscle cell.” These inconsistencies arise partly from the lack of a common naming convention for single-cell annotations and partly from the absence of a standardized reference for hierarchical relationships among cell types. This phenomenon of inconsistent naming leads to incomparable cell labels in single-cell data, affecting dataset integration based on cell types and the construction of cell atlases, as well as the training and evaluation of machine learning methods for cell type annotation across multiple datasets (Osumi-Sutherland *et al.* 2021, Domcke and Shendure 2023). For example, when validating the performance of machine learning models, the inconsistency between the label systems of the training data and external datasets can seriously affect the biological significance of the evaluation metrics, making the evaluation unfair and not objective.

While some institutions and organizations (Bard *et al.* 2005, Diehl *et al.* 2016, Börner *et al.* 2021), such as Cell Ontology, are working toward standardizing cell type nomenclature, they fall short of fully resolving inconsistencies in cell-type labels, particularly in the context of cell annotation. The Cell Ontology database provides standardized cell-type labels for different species to a certain extent; however, it primarily serves as a knowledge repository rather than a user-friendly tool for single-cell annotation and lacks organ-specific focus, especially marker genes information highly relevant to single-cell researchers. Other initiatives also miss the mark by failing to incorporate hierarchical structures of cell types. Most importantly, none of these efforts provide intuitive, user-friendly tools for seamless cell type mapping, making it difficult for users to align their annotations with standardized nomenclature efficiently.

To address these issues, we developed the unified Hierarchical Annotation Framework, uHAF (https://uhaf.unifiedcellatlas.org), which includes a hierarchical cell type tree (uHAF-T) and an easy-to-use tool, the uHAF Mapping Agent (uHAF-Agent), for rapid custom cell type label mapping. uHAF-T provides hierarchical cell type trees for different organs and an accompanying Python package, enabling users to easily unify cell type annotations to the same granularity or trace back to coarser-grained cell types. uHAF-Agent leverages large language models (LLMs) to map diverse aliases and even informal abbreviation of cell types onto corresponding nodes in the uHAF framework. By simplifying the mapping process, uHAF-Agent makes it straightforward to unify cell type labels across datasets. This ease of unification facilitates data integration, boosts machine learning applications, and supports diverse downstream analyses. Additionally, we designed a set of evaluation metrics for cell type annotation based on uHAF. These metrics allow users to choose evaluation granularity and redefine traditional measures such as accuracy and recall in a biologically meaningful way. In summary, uHAF may serve as an essential framework for standardizing cell type annotations, improving data integration, and enabling biologically meaningful evaluations with ease and efficiency.

## 2 Methods

### 2.1 Overview

**Figure 1.**
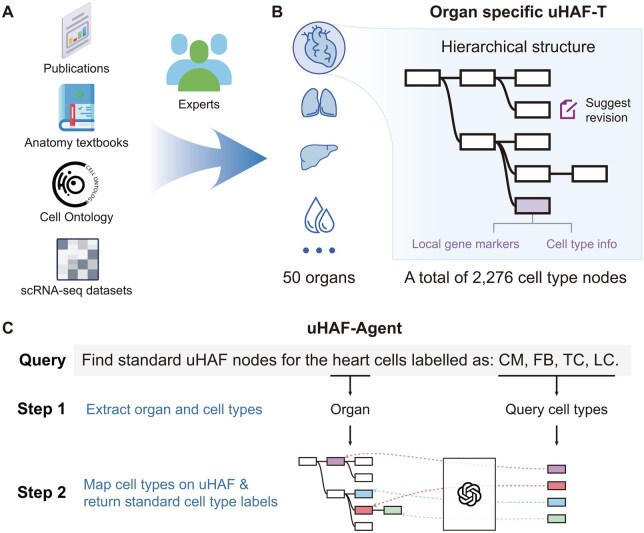
Overview of uHAF. uHAF comprises two main components: the hierarchical cell type trees (uHAF-T) and the LLM-based cell type mapping agent (uHAF-Agent). (A) The curation of uHAF-T, manually created based on anatomical literature, single-cell data-related publications, and the Cell Ontology. (B) uHAF-T covers 50 organs, each with a hierarchical cell type reference tree, totaling 2276 cell type nodes. (C) The workflow of uHAF-Agent. Users can input their cell types and organ in free-text format. uHAF-Agent first extracts the organ and cell types from the input and then uses LLM to map these cell types onto the corresponding organ-specific nodes in uHAF-T.

uHAF consists of two components: organ-specific hierarchical cell type trees (uHAF-Ts) and a cell type mapping agent (uHAF-Agent) that projects custom cell type labels onto uHAF-T (Fig. 1). We provide 50 organ-specific uHAF-Ts, encompassing 2276 cell type nodes and 836 gene markers. Within uHAF-T, the cell types of each organ form a hierarchical tree based on “is_a” relationships, allowing each cell type label to easily find its ancestor nodes. uHAF-Agent is a large language model-based tool that takes users’ customized cell type lists as input and accurately maps each cell type label to the corresponding organ-specific cell type nodes on uHAF-T. With uHAF-Agent, researchers can easily, quickly, and accurately unify the labels of their datasets with the uHAF nodes. These two functionalities are integrated into the uHAF web server.

### 2.2 uHAF-T

uHAF-T includes standardized cell type trees for 50 organs, manually created based on anatomical literature, single-cell data-related publications, and Cell Ontology, as described in Chen *et al.* (2022b). Among these organs, the uHAF-Ts for the heart, lung, liver, blood, bone marrow, and intestine have been thoroughly refined by experts and are relatively complete, while those for other organs may be less comprehensive. In addition to the hierarchical organization of cell types, uHAF-T also provides local markers to distinguish cell types from their sibling nodes.

The uHAF-T can be browsed on the uHAF website’s uHAF-T page. After selecting an organ, the cell types are displayed in a hierarchical structure. By clicking on a specific cell type, we provided two modules: the cell type description module and the local marker module. The cell type description module displays a detailed description of the selected cell type. The local marker module shows gene markers that can be used to identify and differentiate that cell type from its sibling nodes, which can help manually annotate cell types.

uHAF-T can be directly downloaded for local usage or accessed through the uHAF Python package (https://pypi.org/project/uhaf/). The uHAF package additionally provides an ancestor tracing function, allowing users to unify cell types of varying granularity to a consistent coarser granularity. This feature can be useful for dataset integration or annotation algorithm evaluation.

Specifically, to accommodate the continuously growing and updating repertoire of cell types, the uHAF-T webpage offers a revision feature with a user-friendly interactive design. Researchers can efficiently contribute to the database by supplementing or modifying cell types or local markers directly via the website. uHAF will continually be updated and refined by leveraging collective expertise and knowledge so that it is scalable to future accommodate future research.

### 2.3 uHAF-Agent

uHAF-Agent is an agent developed based on OpenAI’s GPT-4 (OpenAI *et al.* 2024) API and is designed to map non-standardized cell type labels from user datasets onto the nodes of uHAF-T, making differently annotated datasets comparable. It takes the unique cell type labels as the input prompt, and outputs pairs of custom labels and their corresponding uHAF nodes. The mapping is conducted based on the semantic information of the custom labels as well as biological knowledge and natural language processing ability of GPT-4.

Users can access uHAF-Agent on the uHAF website’s “uHAF Mapping by custom labels” page. By selecting an organ and entering a cell type list in free text format into the input box, uHAF-Agent returns a dictionary which maps each element in the list to the corresponding uHAF node. The input supports various cell type abbreviations, aliases, special symbols, and even text containing typos. By clicking the “Show on uHAF-T” button, users can view the positions of these cell types within the uHAF-T interface. Users can utilize the results to quickly unify all annotation names in their datasets. Users can further validate or refine the mapping results with local markers using the “uHAF Mapping by marker genes” function on the website.

## 3 Application cases of uHAF

### 3.1 uHAF-Agent is accurate in harmonization of custom labels with uHAF nodes

It is necessary to unify labels across different datasets or align model outputs with dataset labels in various scenarios, such as integrating datasets that require cell type labels [e.g. methods like scANVI (Virshup *et al.* 2023, Xu *et al.* 2023)] and evaluating annotation methods on external datasets. In these contexts, uHAF-Agent enables rapid unification of nomenclature by mapping cell types onto uHAF-T.

To validate the accuracy of uHAF-Agent’s mapping, we conducted six experiments covering three organs—liver (Han *et al.* 2020, Andrews *et al.* 2022, Guilliams *et al.* 2022), lung (Vieira Braga *et al.* 2019, Deprez *et al.* 2020, Sikkema *et al.* 2023), and heart (Chen *et al.* 2022a, The Tabula Sapiens Consortium 2022, Simonson *et al.* 2023)—using three datasets for each organ. We used expert mapping results as the ground truth to calculate the accuracy of uHAF-Agent’s mappings. The original annotations in these scenarios included very abbreviated labels, making the task challenging. For example, a particular cell type Macrophage might be labeled as “Macro” in one dataset and “M∅” in another.

Across the six datasets, uHAF-Agent achieved an average mapping accuracy of 98%. The mapping results (Supplementary Figs S1–S3) illustrate that uHAF-Agent can accurately harmonize custom labels to standard uHAF nodes, regardless of how complex or abbreviated the custom labels are.

### 3.2 uHAF enhances biologically meaningful method evaluation

Using external datasets is a straightforward way to validate the generalization ability of models. However, directly evaluating annotation models on external datasets faces the challenge that the dataset labels and the model outputs often do not match. This mismatch leads to inaccurate metrics and can compromise the biological significance of the evaluation. This problem can be resolved by mapping both the outputs and the labels onto the nodes of uHAF-T. Additionally, the hierarchical nature of uHAF-T facilitates convenient evaluation of cell type annotations at coarser granularity levels.

We used the evaluation of CellTypist (Domínguez Conde *et al.* 2022) on an external lung dataset (Deprez *et al.* 2020) as a case study to demonstrate how uHAF enhances model evaluation accuracy and biological relevance. The lung dataset contains 30 000 cells annotated with 13 cell types. The original annotations of these cells are referred to as *label*, and the corresponding model prediction results are pred. After mapping using uHAF-Agent, the results are *label_uhaf* and *pred_uhaf*. Based on *label_uhaf*, *pred_uhaf*, and uHAF-T, we also obtained coarser-grained hierarchical labels: *label_uhaf_level1*, *label_uhaf_level2*, *pred_uhaf _level1*, and *pred_uhaf_level2*. Using these pairs of labels and predictions, we calculated the model’s accuracy, recall, and F1 score.

The results show that using the original labels and predictions, the accuracy is only 0.09, whereas after uHAF mapping, the accuracy increases to 0.70. This improvement is because directly calculating metrics with the original labels leads to false negatives; e.g. “Basal” and “Basal cell” are considered mismatches, which is not biologically meaningful. The uHAF mapping eliminates this issue by harmonizing the labels. Furthermore, using level 2 and level 1 coarse-grained cell types, the accuracy reaches 0.72 and 0.94, respectively. This indicates that the model performs well in classifying cell types at coarser granularity levels. Recall and F1 scores exhibit similar trends (see Supplementary Fig. S4).

## 4 Discussion

uHAF exhibits a hierarchical structure that aligns with the characteristics of single-cell labels, enabling data integration, cell atlas construction, and more effective evaluation metrics for assessing model performance. In single-cell biology, cell types are naturally organized in hierarchies—e.g. from broad categories like “immune cells” down to specific subtypes like “CD4+ T helper cells.” However, single-cell datasets are generally only labeled with fine-grained cell types. For this reason, specialists often play important roles in these studies. For instance, during the development of the HLCA lung atlas, experts were assembled to perform hierarchical labeling of multi-source data for integration (Sikkema *et al.* 2023). Now, by using uHAF-T and mapping fine cell types with uHAF-Agent, users can efficiently map fine cell types and obtain hierarchical labels. uHAF enables evaluation metrics to go beyond exact label matching, incorporating biological relationships between cell types. This approach allows for a more nuanced and biologically meaningful assessment of model performance by measuring how closely a predicted cell type aligns with the true cell type within the hierarchy. Additionally, the flexible granularity of uHAF allows users to adjust the level of specificity based on their application needs. For instance, coarser granularity may be more suitable for data integration, while broader categories can enhance zero-shot model evaluation. Conversely, fine-grained labels are more appropriate for evaluating models that have undergone task-specific fine-tuning.

Mapping cell type labels is inherently challenging, as it involves mapping from an open domain of diverse and sometimes ambiguous labels to a finite set of standardized nodes. Currently, with the assistance of large language models, this task can be accurately accomplished. This represents a new contribution of LLMs to the field of biology, enhancing the utility of cell type references. Besides, the marker genes included in uHAF can further validate cell type mapping results and even facilitate handling of cell clusters with non-specific labels. uHAF-Agent can assist researchers who specialize in machine learning but may lack a strong biological background to achieve more accurate and efficient cell type label mapping and comparison.

uHAF-T offers several distinct advantages over Cell Ontology, particularly in the context of single-cell annotation. uHAF-T emphasizes cell types and marker genes commonly used in single-cell research, including key marker genes not available in Cell Ontology. Unlike the general structure of Cell Ontology, uHAF-T is organized by organs, providing a more intuitive and accessible framework for researchers studying specific tissues. Additionally, uHAF-T is closely integrated with expression data via linkage with hECA (Chen *et al.* 2022b), enabling users to visualize cell distributions across extensive single-cell atlases. As Cell Ontology serves as a crucial reference for cell biology, our website assigns Cell Ontology identifiers to most of the cell types included in uHAF-T to ensure compatibility and traceability.

Currently, uHAF-T is not yet complete and requires further contributions for corrections and improvements. We are open to collaboration and encourage more people to participate in this endeavor. By working together, we can expand and refine uHAF-T, ensuring it remains a comprehensive and up-to-date resource that benefits the single-cell research community.

## Supplementary Material

btaf149_Supplementary_Data

## Data Availability

The data underlying this article can be found on the Gene Expression Omnibus public database under accession number GSE192742, GSE185477, GSE201333, GSE134355, GSE143868, on European Genome–phenome Archive, EGAS00001001755, and on cellxgene (https://cellxgene.cziscience.com/collections/6f6d381a-7701-4781-935c-db10d30de293).

## Code availability

The code for uHAF package and the experiments in this article is available at https://github.com/SuperBianC/uhaf and permanently archived at Zenodo with DOI https://doi.org/10.5281/zenodo.15022782.
