# Supplementary material for: uHAF: a unified hierarchical annotation framework for cell type standardization and harmonization
Source: Bioinformatics. 2025 Apr 2;41(4):btaf149. doi: 10.1093/bioinformatics/btaf149 (PMC12002906; doi:10.1093/bioinformatics/btaf149)
Supplement: btaf149_Supplementary_Data [file btaf149_supplementary_data.docx]

**Supplementary Figures**


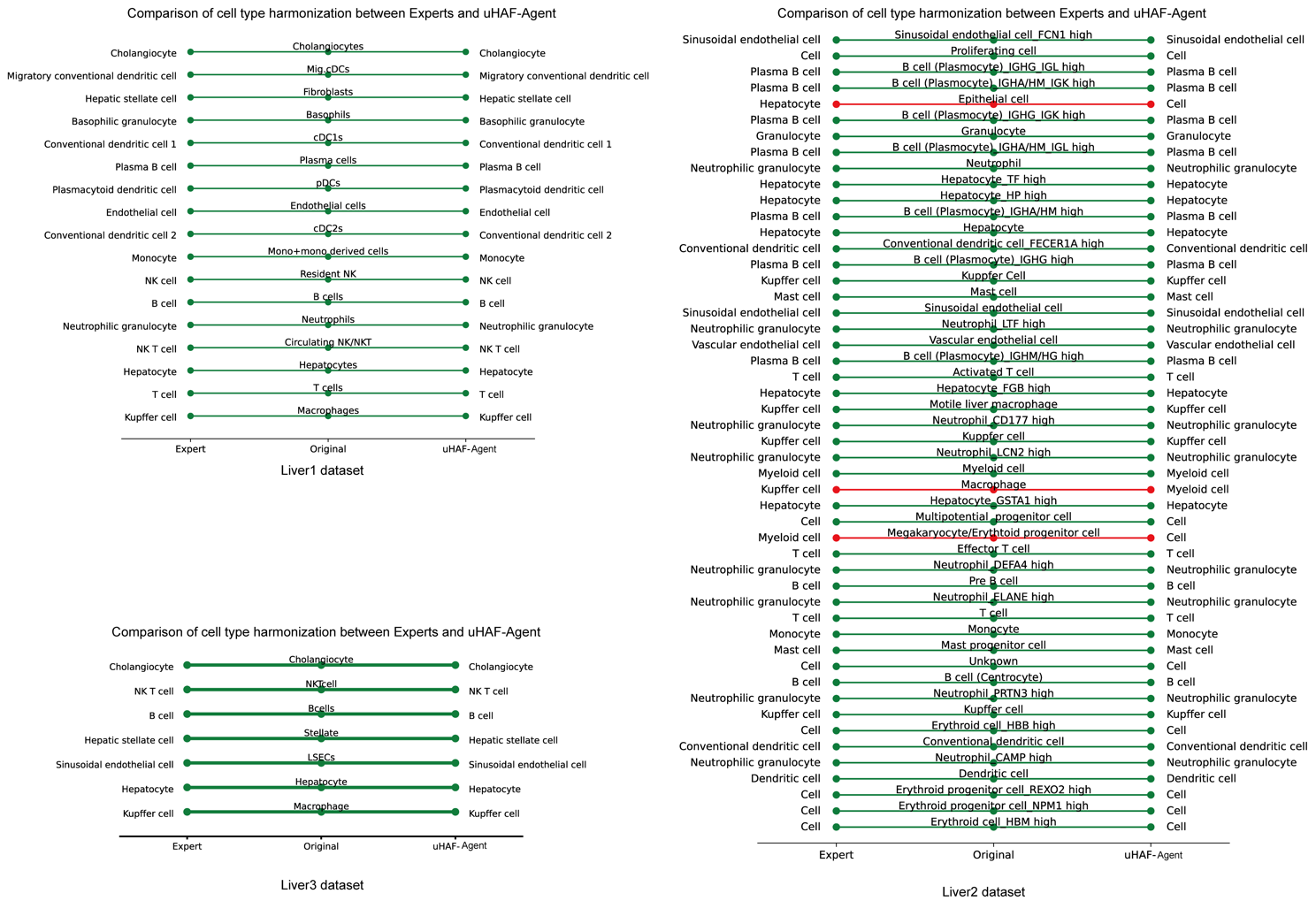


**Figure S1. Mapping results of uHAF_GPT on three liver datasets.** In each subfigure, the nodes in the middle column are the original annotations of the dataset. The nodes in the left column are the uHAF nodes mapped manually by experts, while the nodes in the right column are the uHAF nodes mapped by uHAF-Agent for the corresponding annotations. Green horizontal lines indicate that the uHAF-Agent mapping results are consistent with the expert mapping; red lines indicate inconsistency.


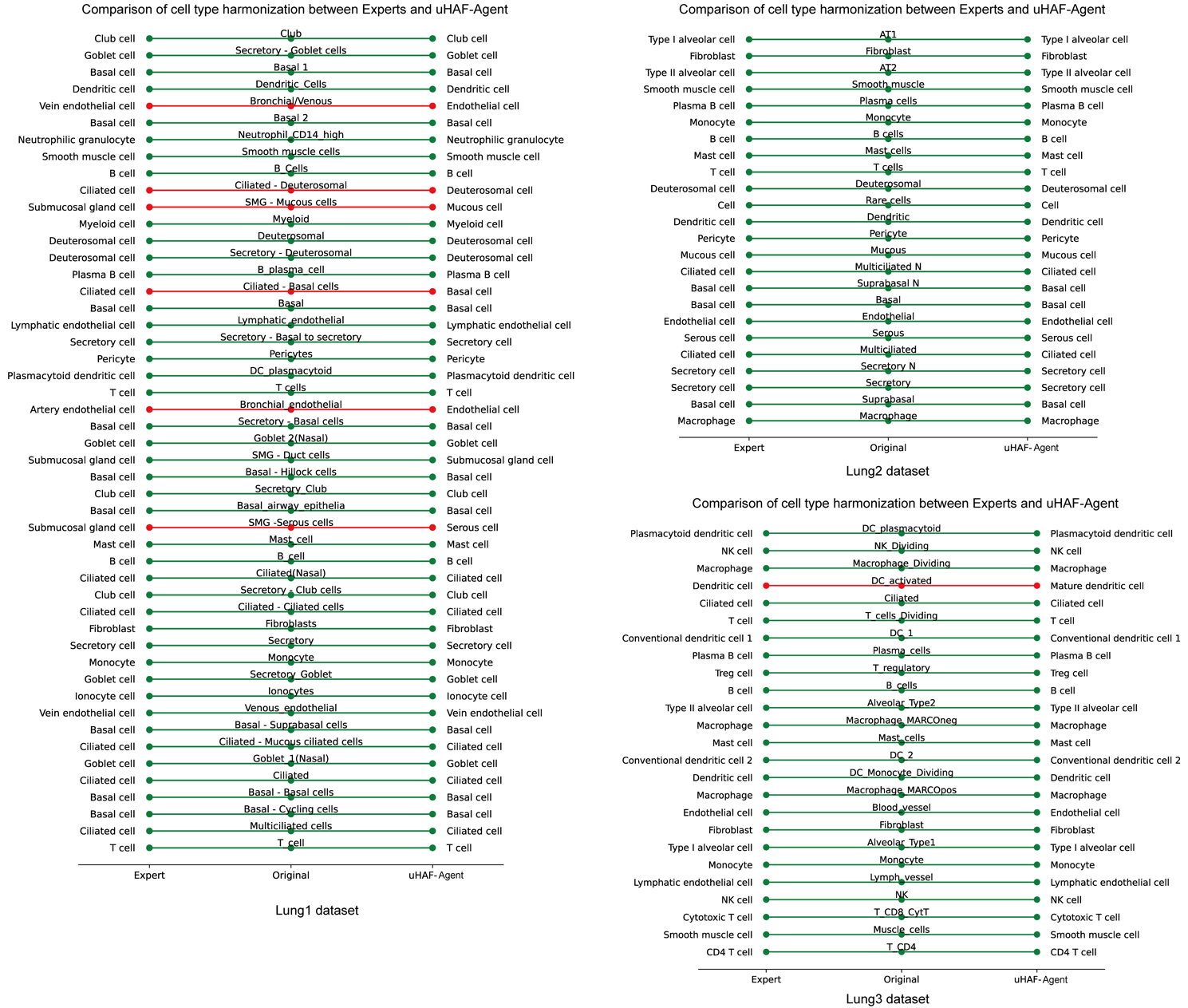


**Figure S2. Mapping results of uHAF_GPT on three lung datasets.** In each subfigure, the nodes in the middle column are the original annotations of the dataset. The nodes in the left column are the uHAF nodes mapped manually by experts, while the nodes in the right column are the uHAF nodes mapped by uHAF-Agent for the corresponding annotations. Green horizontal lines indicate that the uHAF-Agent mapping results are consistent with the expert mapping; red lines indicate inconsistency.


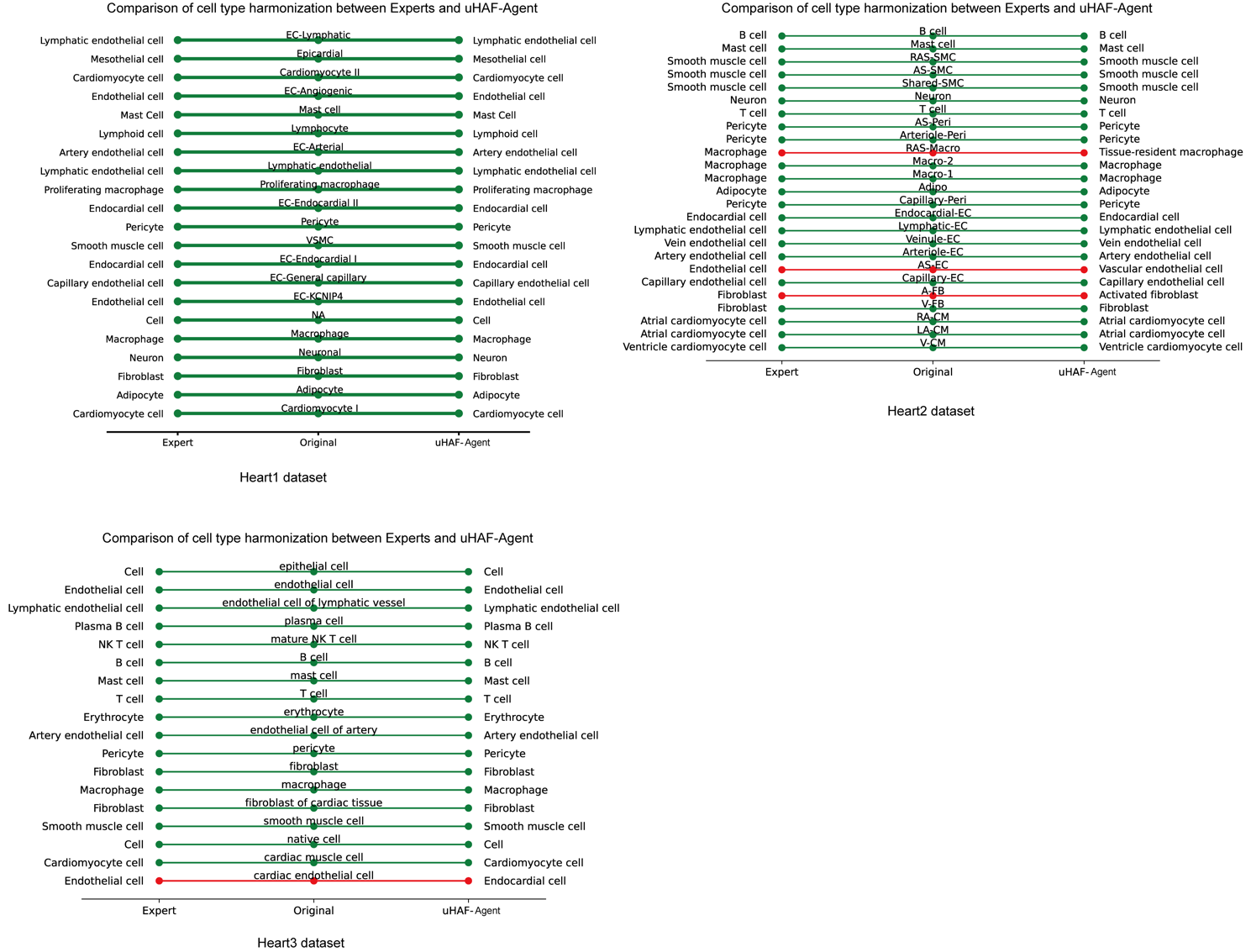


**Figure S3. Mapping results of uHAF_GPT on three heart datasets.** In each subfigure, the nodes in the middle column are the original annotations of the dataset. The nodes in the left column are the uHAF nodes mapped manually by experts, while the nodes in the right column are the uHAF nodes mapped by uHAF-Agent for the corresponding annotations. Green horizontal lines indicate that the uHAF-Agent mapping results are consistent with the expert mapping; red lines indicate inconsistency.

**
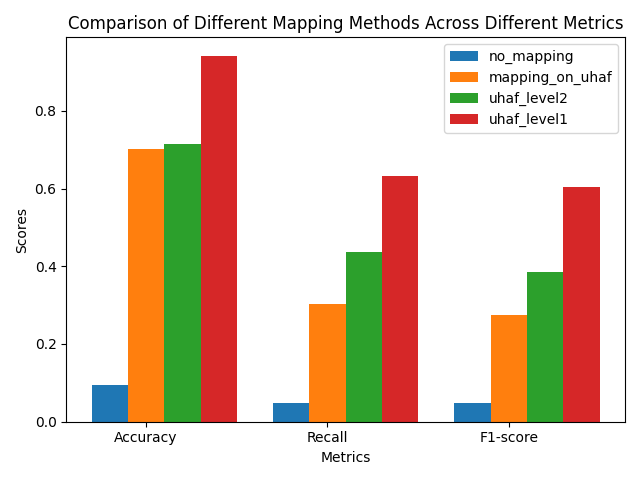
**

**Figure S4. Evaluation results of CellTypist with different mapping strategies.** *No_mapping* refers to the evaluation metrics calculated using the original labels and the original model predictions without any mapping. *Mapping_on_uhaf* involves mapping both the labels and predictions onto uHAF-T using uHAF-Agent. *uhaf_level2* represents the metrics after mapping onto uHAF-T and then extracting the second level of coarser granularity. *uhaf_level1* shows the results at the coarsest granularity after mapping onto uHAF-T.
